# Does Daytime Sleepiness Moderate the Relationship Between Working Memory and Academic Performance in Schoolchildren? A Pilot Study

**DOI:** 10.3390/clockssleep7040057

**Published:** 2025-10-08

**Authors:** Sergey Malykh, Valeriia Demareva

**Affiliations:** 1 Developmental Behavioral Genetics Laboratory, Psychological Institute of Russian Academy of Education, 125009 Moscow, Russia; malykhsb@mail.ru; 2 Cyberpsychology Laboratory, Faculty of Social Sciences, Lobachevsky State University of Nizhny Novgorod, 603950 Nizhny Novgorod, Russia

**Keywords:** working memory, daytime sleepiness, academic achievement, cognitive fatigue, adolescent learning, school performance, sleep hygiene, visuospatial tasks

## Abstract

Academic performance in adolescence is influenced by both cognitive capacity and physiological factors such as sleepiness. However, the interaction between these dimensions remains understudied. This pilot study examined whether daytime sleepiness moderates the relationship between working memory and academic achievement in a sample of 601 schoolchildren aged 11 to 17 years. Participants completed a digital visuospatial working memory task and self-reported their daytime sleepiness using the Pediatric Daytime Sleepiness Scale (PDSS). Academic performance was assessed through official grades in Mathematics, Language, and Literature. Regression analyses showed that working memory (total score and average reaction time) and daytime sleepiness were independent predictors of academic performance. These findings support our hypotheses that cognitive and physiological factors each contribute to school success. However, no significant moderation effects were found in the full sample. Subgroup analyses revealed that working memory predicted academic outcomes only among students with normal sleepiness levels, whereas in high-sleepiness students, cognitive predictors lost significance and PDSS scores emerged as the dominant predictor. These results suggest that elevated daytime sleepiness can undermine the positive impact of working memory on academic performance. The findings highlight the importance of assessing both cognitive skills and physiological readiness when evaluating students. They also suggest that sleep-focused interventions may improve learning outcomes, especially during adolescence.

## 1. Introduction

Academic achievement during school years is a strong predictor of children’s future educational and occupational outcomes [1]. While many factors shape academic success, two domains have emerged as particularly influential: cognitive capacities (e.g., working memory) and sleep-related functioning (e.g., daytime sleepiness). Working memory, as a core cognitive system responsible for the active maintenance and manipulation of information [2], plays a central role in supporting classroom learning and problem-solving [3]. At the same time, sleep—and more specifically, daytime sleepiness resulting from poor or insufficient sleep—is known to compromise alertness and cognitive functioning, with direct consequences for academic performance [3,4,5].

Despite growing evidence in both areas, relatively few studies have examined how cognitive and physiological factors may operate together to influence school outcomes. To address this gap, we examined whether daytime sleepiness moderates the relationship between working memory and academic performance in schoolchildren.

### 1.1. Working Memory and School Performance

Working memory (WM) refers to a limited-capacity system that supports the temporary storage and mental manipulation of information. It underlies complex cognitive activities such as reading comprehension, mathematical reasoning, and learning from instruction [2]. A large body of research has demonstrated moderate to strong correlations between WM and performance in key academic domains, including mathematics, language, and reading [3,6]. Indeed, some findings suggest that WM may be as important as general intelligence in explaining academic differences among children [7,8].

WM is also a developmental construct: it improves significantly with age during childhood and adolescence, in parallel with the maturation of the prefrontal cortex [9]. As academic tasks grow more complex at higher grade levels, the role of WM may become increasingly critical [10]. Notably, cross-cultural research has shown that WM is a universal predictor of achievement [11], further underscoring its relevance in diverse educational contexts.

In the present study, visuospatial WM was assessed with the Corsi Block-Tapping Test. This task captures short-term storage and executive planning in the visuospatial domain, processes that have been repeatedly linked to mathematics performance and broader academic achievement [12,13]. The Corsi Block-Tapping Test has been widely applied across international samples, demonstrates good reliability, and is well suited for administration in classroom settings.

### 1.2. Daytime Sleepiness and Learning

Adequate sleep is essential for memory consolidation, attentional control, and executive functioning—abilities fundamental for learning [4]. Inadequate or disrupted sleep can result in daytime sleepiness, which in turn reduces children’s alertness and classroom engagement [14,15]. Meta-analytic studies confirm that greater daytime sleepiness is associated with lower school grades [5], and adolescents, in particular, are at high risk due to chronic sleep restriction [16].

Large-scale reports show that more than 70% of high school students sleep less than the recommended number of hours on school nights, i.e., 9–12 h for school-aged children and 8–10 h for adolescents [17]. This widespread sleep deprivation suggests that cognitive skills alone may not be sufficient to support academic performance when students are not adequately rested. Importantly, the prevalence and impact of sleepiness increase with age, as sleep duration declines during adolescence due to biological and social factors [18].

In addition to sleep deprivation and subjective sleepiness, related circadian factors such as timing of instructional sessions, wake time, and daytime napping can also affect learning and performance. For instance, Mehta [19] showed that university students’ academic performance was associated with the timing of teaching sessions and napping behavior: short naps (≈20 min) and morning sessions were linked to higher scores, whereas late-day sessions and longer naps predicted lower performance. Therefore, circadian rhythm and sleep behaviors may influence achievement in ways that are distinct from, but complementary to, daytime sleepiness as a state of insufficient alertness.

### 1.3. Interplay Between Working Memory and Sleepiness

While WM and sleepiness have both been independently linked to academic outcomes, their potential interaction remains understudied. One possibility is that daytime sleepiness partially moderates the impact of WM on performance. That is, students with lower WM may take longer to process and complete cognitive tasks, making them more vulnerable to mental fatigue and later sleepiness during the school day—an effect that could further diminish academic outcomes. Alternatively, stronger WM may buffer the cognitive effects of mild sleepiness, supporting performance despite drowsiness [1].

Moreover, as children mature, changes in both WM and sleep behavior could lead to age-related variation in how these factors influence learning [9,16]. The developmental divergence—where WM improves and sleep quality worsens with age—raises questions about how the relationship between cognitive resources and sleep-related functioning may shift over time.

### 1.4. The Present Study

This pilot study explores whether daytime sleepiness moderates the relationship between working memory and academic performance in schoolchildren aged 11 to 17. Unlike prior studies that consider WM and sleep independently, our design allows for an integrated analysis of their combined and potentially sequential effects. We assess working memory using two indicators, accuracy (total score) and average reaction time from a standardized visuospatial task, and quantify daytime sleepiness via the Pediatric Daytime Sleepiness Scale (PDSS). Academic outcomes include subject-specific grades in Mathematics, Russian Language, and Literature, as well as a composite Average Grade.

The study addresses the following research question:

Does daytime sleepiness moderate the relationship between working memory and academic performance?

Based on the literature, we hypothesize the following:

**H1:**  *Working memory will be positively associated with academic outcomes.*

**H2:**  *Daytime sleepiness will be negatively associated with academic outcomes.*

**H3:**  *Daytime sleepiness will partially moderate the relationship between working memory and academic achievement.*

By combining cognitive and physiological predictors within a single statistical framework, this study contributes to a more nuanced understanding of how internal resources and behavioral health jointly influence learning. The results have potential implications for both cognitive assessment practices and sleep-related interventions in school settings.

## 2. Results

### 2.1. Descriptive Statistics

The final sample included 601 students aged 11 to 17 years. A total of 310 participants were female (52%), and 291 were male (48%). All participants had complete academic records in Russian language (Language), Mathematics, and Literature, as well as valid cognitive and sleepiness assessments. Table 1 presents the distribution of participants by school level and gender.

Descriptive statistics were calculated for the primary variables, including academic performance (Average Grade and subject-specific scores), working memory (CTB Total Score and average reaction time for correct answers), and daytime sleepiness (PDSS total score). As shown in Table 2, the mean average grade was 4.05 (SD = 0.58), while the average PDSS score was 14.07 (SD = 6.45), indicating moderate levels of daytime sleepiness in the sample.

The average and median grades were relatively high (all medians = 4 on a 2–5 scale), reflecting typical grading practices in the participating schools. Descriptive statistics of academic outcomes and task performance are presented separately for middle and high school students in Supplementary Table S2. High school students reported higher daytime sleepiness (PDSS: 14.92 vs. 13.51) and higher CTB Total Score (5.21 vs. 4.39). Academic grades also tended to be higher among high school students (e.g., Mathematics: 4.14 vs. 3.81; Language: 3.98 vs. 3.89), whereas Literature grades were comparable across levels (4.29 vs. 4.29).

To preliminarily examine the relationships among key variables, Pearson correlation coefficients were calculated between academic performance indicators (Average Grade, Russian Language, Mathematics, and Literature grades), cognitive measures (CTB Total Score and Average Correct Response Time), and daytime sleepiness (PDSS total score). The results are presented in Table 3.

Correlation analysis revealed several significant associations among the core variables (Table 3). Average Grade demonstrated strong positive correlations with individual subject grades in Russian language (*r* = 0.89, *p* < 0.001), Mathematics (*r* = 0.85, *p* < 0.001), and Literature (*r* = 0.84, *p* < 0.001), reflecting internal consistency of academic performance. Working memory (CTB Total Score) showed a modest but significant positive correlation with both Average Grade (*r* = 0.12, *p* = 0.004) and mathematics grade (*r* = 0.14, *p* = 0.001), supporting its role in academic achievement. By contrast, daytime sleepiness (PDSS) was negatively associated with all academic indicators, with the strongest correlation observed for Average Grade (*r* = −0.11, *p* = 0.005), suggesting that increased sleepiness may impair overall academic functioning. Reaction time (CTB Average RT) was negatively related to mathematics performance (*r* = −0.10, *p* = 0.017), indicating that slower cognitive processing may be associated with lower achievement in numeracy.

### 2.2. Moderation Analysis: Does Daytime Sleepiness Alter the Effect of Working Memory on Academic Achievement

To test whether daytime sleepiness moderates the relationship between working memory and academic achievement (Average, Mathematics, Russian language, and Literature grades), multiple linear regression models were conducted including the interaction terms between sleepiness (PDSS) and two indicators of working memory performance: total score (CTB Total Score) and average reaction time (CTB Average RT) on the Corsi Block-Tapping Test (CTB). The models also controlled for sex and school level.

The model was statistically significant, *F*(7, 593) = 11.26, *p* < 0.001, and explained approximately 11.7% of the variance in Average Grade (*R*^2^ = 0.117, adjusted *R*^2^ = 0.107).

Table 4 presents the results.

As shown in Table 4, CTB Total Score (*B* = 0.06, *SE* = 0.02, *p* = 0.010), PDSS (*B* = −0.10, *SE* = 0.02, *p* < 0.001), and CTB Average RT (*B* = −0.06, *SE* = 0.02, *p* = 0.008) were significant predictors of Average Grade. Female sex was associated with higher grades (*B* = 0.33, *SE* = 0.05, *p* < 0.001), while school level showed a marginal effect (*B* = 0.09, *SE* = 0.05, *p* = 0.052). The interaction terms between PDSS and the working memory indicators were not statistically significant (*p* = 0.772 and *p* = 0.215, respectively). Adding a CTB Total × CTB Average RT interaction did not improve fit, and the term was not significant for any outcome (|*B*| < 0.03; 95% *CI*s spanning zero).

The overall model was statistically significant, *F*(7, 593) = 12.09, *p* < 0.001, and explained approximately 12.5% of the variance in Mathematics grades (*R*^2^ = 0.125, adjusted *R*^2^ = 0.115). See Table 5 for details.

Both CTB Total Score (*B* = 0.07, *SE* = 0.03, *p* = 0.013) and CTB Average RT (*B* = −0.10, *SE* = 0.03, *p* = 0.001) significantly predicted mathematics grades. Daytime sleepiness (PDSS) had a negative effect (*B* = −0.12, *SE* = 0.03, *p* < 0.001). Sex (female) and higher school level also positively predicted mathematics grades (both *p* < 0.001). Neither interaction term was significant.

The regression model was statistically significant, *F*(7, 593) = 10.25, *p* < 0.001, explaining approximately 10.8% of the variance in Russian language grades (*R*^2^ = 0.108, adjusted *R*^2^ = 0.097)—see Table 6.

In predicting Russian language grades, CTB Total Score (*B* = 0.07, *SE* = 0.03, *p* = 0.008) and CTB Average RT (*B* = −0.07, *SE* = 0.03, *p* = 0.008) were significant positive and negative predictors, respectively. PDSS also negatively predicted language grades (*B* = −0.09, *SE* = 0.03, *p* < 0.001). Sex (female) was associated with higher grades (*B* = 0.38, *SE* = 0.05, *p* < 0.001), while school level did not reach significance (*p* = 0.554). Interaction terms were non-significant.

To evaluate whether daytime sleepiness moderates the relationship between working memory and performance in Literature, we conducted a multiple linear regression analysis. The model included interaction terms between PDSS (daytime sleepiness) and two indicators of working memory—CTB Total Score and CTB Average Reaction Time—while controlling for sex and school level.

The model was statistically significant, *F*(7, 593) = 5.76, *p* < 0.001, explaining approximately 6.4% of the variance in Literature Grade (*R*^2^ = 0.064, adjusted *R*^2^ = 0.053)—see Table 7.

For Literature Grade, only PDSS (*B* = −0.09, *SE* = 0.03, *p* < 0.001) and sex (female; *B* = 0.28, *SE* = 0.05, *p* < 0.001) were significant predictors. CTB Total Score, CTB Average RT, school level, and interaction terms were not significant.

**Figure 1:**
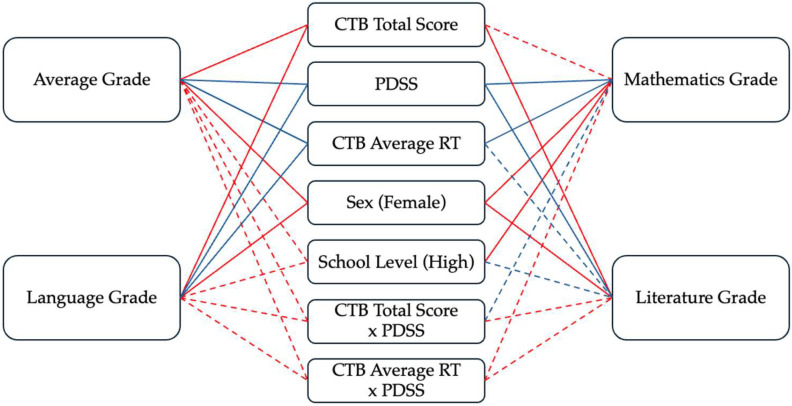
Path diagram of the moderation models predicting academic performance (Average Grade, Mathematics, Russian Language, and Literature). CTB = Corsi Block-Tapping Test; RT = reaction time; PDSS = Pediatric Daytime Sleepiness Scale. Solid lines represent significant paths (*p* < 0.05), dashed lines represent nonsignificant paths. Blue lines indicate negative associations, red lines indicate positive associations.

Figure 1 presents a path diagram of the moderation models, summarizing the effects of working memory (CTB Total Score and CTB Average RT), daytime sleepiness (PDSS), and covariates on academic outcomes. Solid lines represent significant paths, while dashed lines indicate nonsignificant effects.

### 2.3. Moderation Analysis: Does the Moderating Role of Daytime Sleepiness Differ by Its Severity?

To further explore whether the moderating role of daytime sleepiness differs by its severity, we performed separate regression models for students with normal (<16; *N* = 362) and high (≥16; *N* = 239) PDSS scores.

#### 2.3.1. Baseline Differences by Daytime Sleepiness Group

The high-sleepiness group was significantly older (*M* = 14.45, *SD* = 1.96) than the normal-sleepiness group (*M* = 13.99, *SD* = 2.00), Welch’s *t*(517) = −2.76, *p* = 0.006, Cohen’s *d* = −0.23. Sex distribution also differed, with a higher proportion of females in the high-sleepiness group (61.9% vs. 44.8%), χ^2^(1) = 16.32, *p* < 0.001, Cramer’s *V* = 0.17. In addition, school level was associated with sleepiness status, with more high-sleepiness students enrolled in high school, χ^2^(1) = 7.30, *p* = 0.007, Cramer’s *V* = 0.11.

CTB Total Score did not differ significantly between groups (normal: *M* = 4.77, *SD* = 1.95; high: *M* = 4.63, *SD* = 1.95), Welch’s *t*(510) = 0.86, *p* = 0.393, Cohen’s *d* = 0.07. By contrast, CTB Average RT was shorter in the high-sleepiness group (lower RT values; normal: *M* = 4763.69 ms, *SD* = 1526.33; high: *M* = 4488.61 ms, *SD* = 1482.72), Welch’s *t*(520) = 2.20, *p* = 0.028, Cohen’s *d* = 0.14. See Table 8.

Because both age and school level were related to sleepiness and capture overlapping developmental variance, only school level was included as a covariate in subsequent regression analyses to avoid collinearity and model complexity. CTB Total Score and CTB Average RT were retained as working memory predictors in all models in line with the study hypotheses.

#### 2.3.2. Moderation Analysis by Daytime Sleepiness Severity

In students with normal daytime sleepiness, the model was statistically significant, *F*(7, 354) = 4.68, *p* < 0.001, explaining 8.5% of the variance in average grades (*R^2^* = 0.085, adjusted *R*^2^ = 0.067). CTB Total Score significantly predicted Average Grade (*B* = 0.10, *SE* = 0.05, *p* = 0.027), and CTB Average RT showed a marginally negative effect (*B* = −0.07, *SE* = 0.04, *p* = 0.098). PDSS was not a significant predictor in this group (*p* = 0.300). The interaction terms were not significant (*p* > 0.2). Sex had a strong effect, with girls outperforming boys (*p* < 0.001).

For students with high daytime sleepiness, the model was also significant, *F*(7, 231) = 6.77, *p* < 0.001, accounting for 17.0% of the variance (*R^2^* = 0.170, adjusted *R*^2^ = 0.145). PDSS had a significant negative effect on grades (*p* = 0.016). Neither CTB Total Score (*p* = 0.117) nor reaction time (*p* = 0.271) were significant. Again, interaction effects were non-significant. Sex remained a significant predictor (*p* < 0.001). See Table 9.

The model for students with normal daytime sleepiness was statistically significant, *F*(7, 354) = 6.04, *p* < 0.001, explaining 10.7% of the variance in Mathematics Grade (*R*^2^ = 0.107, adjusted *R*^2^ = 0.089). In the normal sleepiness group, CTB Total Score (*B* = 0.12, *SE* = 0.06, *p* = 0.045) and CTB Average RT (*B* = −0.11, *SE* = 0.06, *p* = 0.046) significantly predicted Mathematics Grade. However, PDSS itself and all interaction effects were non-significant.

For students with high daytime sleepiness, the model remained significant, *F*(7, 231) = 5.65, *p* < 0.001, accounting for 14.6% of the variance (*R*^2^ = 0.146, adjusted *R*^2^ = 0.120). None of the cognitive predictors reached statistical significance, no interactions effects were observed. However, sex and school level remained significant predictors. See Table 10.

For subsample with normal daytime sleepiness, the model was statistically significant, *F*(7, 354) = 5.08, *p* < 0.001, and explained 9.1% of the variance in Russian language grades (*R*^2^ = 0.091, adjusted *R*^2^ = 0.073). CTB Total Score significantly predicted performance in Russian among students with normal sleepiness. No interaction effects were found.

The model for students with high sleepiness level was also statistically significant, *F*(7, 231) = 6.42, *p* < 0.001, explaining 16.3% of the variance (*R*^2^ = 0.163, adjusted *R*^2^ = 0.137). Only PDSS and sex were significant predictors, while the effect of working memory was non-significant. See Table 11.

The model did not reach statistical significance, *F*(7, 354) = 1.65, *p* = 0.121, and explained only 3.2% of the variance in Literature grades (*R*^2^ = 0.032) for students with normal sleepiness level. This suggests that, in the group of students with normal sleepiness, the predictors included in the model did not collectively explain a meaningful amount of variance in literature performance. Only sex had a statistically significant effect in this subgroup. Neither working memory performance nor sleepiness levels showed meaningful associations with literature grades.

In contrast, the model for the high-sleepiness group was statistically significant, *F*(7, 231) = 4.53, *p* < 0.001, and explained 12.1% of the variance in literature grades (*R*^2^ = 0.121). However, as with the previous subgroup, most predictors did not reach significance. None of the cognitive predictors or interaction terms reached significance. There was only a marginal effect of PDSS (*p* = 0.068), and once again, sex was a strong predictor, with female students achieving higher grades. See Table 12.

## 3. Discussion

### 3.1. Summary of Main Findings and Hypothesis Testing

The aim of this study was to investigate whether daytime sleepiness moderates the relationship between working memory and academic achievement in school-aged children and adolescents. We tested three main hypotheses: (H1) working memory would positively predict academic performance; (H2) daytime sleepiness would negatively predict academic performance; and (H3) daytime sleepiness would moderate the relationship between working memory and academic achievement. Our findings partially support these hypotheses.

Consistent with H1, working memory—as measured by Corsi Block-Tapping Test (CTB) total score and average reaction time (response latency)—was significantly associated with academic performance across multiple domains, especially in Mathematics and Russian language. These results replicate prior findings that working memory capacity plays a key role in supporting complex academic tasks [3,6]. Similarly, supporting H2, higher levels of daytime sleepiness were consistently associated with lower academic performance, particularly in students with elevated PDSS scores. These results align with existing literature showing that insufficient sleep and reduced alertness can hinder learning and concentration [4,5,14].

Literature grades showed weaker associations with working memory and daytime sleepiness than Mathematics or Language grades. One possible explanation is that performance in literature relies more heavily on crystallized knowledge (e.g., accumulated vocabulary, prior reading experience, cultural knowledge) and long-term memory retrieval, rather than the fluid cognitive resources of online manipulation and maintenance, as tapped by visuospatial WM tasks. According to the Cattell–Horn–Carroll framework, fluid abilities (such as WM and processing speed) are more predictive of mathematics and novel problem-solving, whereas crystallized abilities support tasks requiring comprehension, integration of prior knowledge, and verbal analysis [20,21]. Prior research has also shown that mathematics performance is more dependent on executive functions, while reading and literature tasks often correlate more strongly with verbal comprehension and accumulated knowledge [22,23,24]. Thus, the weaker associations observed for literature may reflect domain-specific cognitive demands that are less sensitive to acute fluctuations in attention and sleepiness.

H3 was not supported in the full sample: daytime sleepiness did not significantly moderate the association between working memory and academic achievement when tested through interaction terms in regression models. This finding might suggest that the two factors act additively rather than interactively. Yet, a closer look at subgroup analyses revealed patterns that suggest a more nuanced dynamic.

Our choice to fit the full, theory-driven model (rather than stepwise hierarchical variants) reflects the a priori hypothesis that each WM facet could show a distinct, PDSS-dependent association with achievement; sensitivity analyses with each WM predictor entered alone yielded the same qualitative conclusions.

### 3.2. Subgroup Analyses as Indirect Evidence of Interaction

Although interaction terms were statistically non-significant in the overall models, subgroup analyses by daytime sleepiness level revealed divergent predictive patterns that offer indirect support for the hypothesized interplay between cognitive and physiological variables.

In the group of students with normal levels of daytime sleepiness, working memory (especially CTB Total Score) significantly predicted academic success, including in mathematics and Russian language. This suggests that in the absence of physiological fatigue, cognitive abilities such as working memory are effectively engaged in learning tasks. By contrast, in the high-sleepiness group, working memory indicators lost predictive power, while sleepiness became the dominant predictor of school performance. This shift suggests a functional suppression effect, where even strong cognitive resources may fail to translate into academic success when students are physiologically compromised.

Such patterns align with models of resource interference, in which cognitive capacity is not simply additive but contingent on the learner’s physiological and motivational state [1]. While not constituting formal statistical moderation, the subgroup divergence indicates that the relationship between working memory and school performance is conditional on levels of daytime alertness—thus echoing the logic of moderation in practice.

Interestingly, this compensatory pattern was especially clear in mathematics, where both working memory measures (total score and average reaction time) predicted performance in the low-sleepiness group but not in the high-sleepiness group. Since math tasks place particularly high demands on mental manipulation and sustained attention, these findings underscore the vulnerability of cognitively demanding domains to physiological fatigue. Literature grades, in contrast, were less consistently associated with working memory or sleepiness, suggesting a possible domain-specific effect, also observed in prior studies [10].

Another key result across both full and subgroup models was the persistent influence of sex, with female students significantly outperforming male peers across subjects. Outperformance of female students across academic subjects aligns with a broader body of research, suggesting that the observed gender gap is multifactorial in nature. Cognitive and behavioral differences, such as higher levels of self-discipline and motivation among girls, have been frequently identified as contributing factors [25]. Socio-cultural expectations and gender socialization may also play a role, with girls often receiving more positive reinforcement for compliance and academic diligence [26]. Additionally, earlier brain development in females could influence learning outcomes during critical academic periods [27]. While beyond the primary scope of this study, this consistent effect aligns with prior educational data and may interact with both sleep behaviors and cognitive strategies—meriting further exploration in future work.

### 3.3. Developmental Implications

The developmental nature of the sample (ages 11–17) is especially relevant. Adolescents experience both an increase in working memory capacity [28] and a decline in sleep quality and duration [9,16]. The findings suggest that as students grow older, the limiting factor for academic performance may shift from cognitive capacity to physiological readiness. In other words, older students may have sufficient cognitive skills but lack the alertness necessary to apply them, particularly if they accumulate sleep debt. Future research should explore these developmental dynamics longitudinally, examining how pubertal status, school start times, and extracurricular demands affect the WM–sleepiness–achievement interplay.

These insights have practical implications for educational policy and school health programs. Interventions that target only cognitive skill development may be insufficient if physiological barriers like chronic sleepiness go unaddressed. Conversely, efforts to improve sleep hygiene might unlock existing cognitive potential, especially among adolescents.

### 3.4. Limitations and Future Directions

Several limitations of this study should be acknowledged.

Academic outcomes: Although we used official school grades, these measures are inherently subjective, potentially influenced by teacher expectations, classroom behavior, and student motivation, rather than reflecting pure academic mastery. In addition, students contributed an unequal number of quarterly grades (one or two), which may have introduced variability into the computation of outcomes. Although we averaged the available grades, this imbalance between grade levels should be taken into account when interpreting the results. Future studies should incorporate standardized tests or curriculum-based assessments as complementary indicators of academic achievement and control for motivational and socio-economic factors that are known to shape performance.

Sleepiness measurement: Daytime sleepiness was measured solely by student self-reports on the Pediatric Daytime Sleepiness Scale (PDSS). These ratings were not corroborated by parents or teachers, and no objective measures (e.g., actigraphy) were included, which raises the possibility of reporting bias. Moreover, we did not collect longitudinal data on the persistence or duration of students’ sleepiness symptoms. Future research should therefore integrate multi-informant approaches (e.g., student, parent, teacher reports) and longitudinal methods such as sleep diaries or actigraphy to enhance the reliability and ecological validity of sleepiness assessment.

Working memory assessment: Working memory was assessed only through a visuospatial task (Corsi Block-Tapping Test). While this measure is reliable and efficient for group testing, it does not capture verbal or other executive aspects of working memory. Future research should therefore include verbal WM tasks and a broader cognitive battery to provide a more comprehensive account of how different WM components contribute to domain-specific academic outcomes, particularly in language and literature.

Sample and design: The study sample was drawn from public schools in Nizhny Novgorod, which may limit generalizability given differences in educational practices and sleep habits across regions and cultures. In addition, the cross-sectional design precludes causal inferences regarding developmental changes. Longitudinal studies—using designs such as cross-lagged panel models or intervention trials—are needed to clarify trajectories and establish causality. Although our sample size provided adequate power to detect moderate interaction effects, the study may have been underpowered to identify smaller effects.

Future directions: Beyond replication in more diverse cultural and educational contexts, future studies should investigate the neurocognitive mechanisms underlying the observed associations. Neuroimaging methods could be employed to examine potential compensatory neural processes. Incorporating pubertal and maturational indicators would further clarify developmental transitions during adolescence. Additionally, clarifying the consistent female advantage in academic outcomes should remain a research priority, exploring biological, cognitive, and socio-cultural explanations. Finally, these findings have practical implications: educational strategies that integrate cognitive development and sleep management into curricula, and interventions tailored to developmental stages, may help optimize learning outcomes.

## 4. Materials and Methods

### 4.1. Participants

The study sample consisted of 639 schoolchildren (52% female, 48% male) aged 11–17 years, attending public schools in Nizhny Novgorod. This study was conducted during the 2024–2025 academic year (November to December 2024), corresponding to the autumn-winter school term in Nizhny Novgorod.

The sample was drawn from four public schools in Nizhny Novgorod. The number of participants per school ranged from 120 to 180. Because our focus was on student-level associations, and preliminary analyses showed no significant between-school differences in academic grades or PDSS scores, school was not included as a factor in the regression models. All schools followed the same regional curriculum, and testing conditions were standardized across sites by using the same trained personnel, procedures, and equipment.

Data underwent quality control procedures to remove outliers. Specifically, reaction times from the working memory tasks were analyzed using the Interquartile Range (IQR) method. Values falling outside 1.5 × IQR (above or below) were identified as outliers and subsequently removed, following the classic Tukey approach. After data cleaning, the final analytic sample comprised 601 observations.

All procedures were conducted in accordance with the ethical standards of the 1964 Helsinki Declaration and its later amendments. Informed consent was obtained from legal guardians of all participants prior to data collection. Children were also informed about the purpose of the study and participated voluntarily. The study was approved by the Ethics Committee of the Psychological Institute of the Russian Academy of Education, protocol No. 2020/4 1 dated 2 April 2020.

All assessments were carried out during scheduled school hours in classroom settings. Students were tested in groups of 10–15 peers under standardized conditions. The tasks were administered by trained study personnel, not schoolteachers, to ensure consistency and reduce potential bias. Testing sessions were scheduled at a consistent time of day (typically between 10:00 a.m. and 12:00 p.m.) across schools to minimize variability due to circadian factors.

### 4.2. Measures

#### 4.2.1. Academic Achievement

Academic performance was assessed based on school grades in three core subjects: Mathematics, Russian Language, and Literature. These data were provided directly by the schools in the form of spreadsheets exported from students’ electronic gradebooks. Grades ranged from 2 to 5; when multiple quarterly grades were available, we computed their average for each subject. Specifically, 325 middle-school students and 217 high-school students contributed one quarterly grade, while 38 middle-school and 21 high-school students contributed two quarterly grades. The detailed distribution is presented in Supplementary Table S1. To obtain a general index of academic success, a composite measure (Average Grade) was computed as the arithmetic mean of the three subject-specific averages.

#### 4.2.2. Working Memory Assessment

Working memory was assessed using a revised and shortened digital version of the Corsi Block-Tapping Test [29], adapted for online administration and validated for Russian speaking schoolchildren [10,28]. Participants viewed a series of blocks on the screen that lit up sequentially in a specific order. Their task was to reproduce the correct sequence by clicking the corresponding blocks with a mouse.

The test began at Level 4, where each sequence consisted of 4 blocks. Each level included two sequences, and the total number of levels ranged from 4 to 9, yielding 18 trials in total. The test progressed to higher levels only if participants correctly completed at least one sequence at the current level. The task was discontinued if both sequences at the same level were reproduced incorrectly.

Before the actual test, participants were given visual instructions and a practice trial of three items, which could be repeated until the task was understood. During the presentation phase, each block glowed for 1 s, with a 1-s pause between glows. Responses were recorded as sequences of block selections and could not be altered once made (i.e., participants could not undo a click).

The test captured two key performance metrics:

1. CTB Total Score: the total number of correctly reproduced sequences.
2. CTB Average Reaction Time (RT): the mean latency for correct responses, recorded automatically by the software.

For analyses we used the CTB Total Score rather than a proportion correct. Accuracy (number of correct reproductions divided by the total number of trials) was computed during preliminary analyses and yielded comparable results; thus, for simplicity we report raw total scores.

#### 4.2.3. Daytime Sleepiness

Daytime sleepiness was assessed with the Pediatric Daytime Sleepiness Scale (PDSS), which has been validated in the Russian population [30]. The scale demonstrated acceptable reliability (Cronbach’s α ≈ 0.78). It consists of eight items rated on a five-point scale from 0 (never) to 4 (always). The total score ranges from 0 to 32, with higher scores indicating greater levels of daytime sleepiness. Participants reported their sleepiness levels, with higher scores indicating greater daytime sleepiness. PDSS scores were further categorized into normal and high with a cutoff of 16 for both sexes [31,32]. Participants completed the questionnaire independently in the classroom; parental confirmation was not obtained, which we acknowledge as a limitation.

### 4.3. Statistical Analysis

All analyses were conducted in R software (2024.04.2 Build 764). Descriptive statistics were calculated for all major variables. Outliers in reaction time data from the working memory task were identified and removed using the interquartile range (IQR) method prior to analysis.

Continuous predictors were z-standardized prior to modelling. Pairwise correlations among predictors were small (|*r*| ≤ 0.12); notably, CTB Total and CTB Average RT correlated only modestly (*r* = 0.11, *p* < 0.01). Variance-inflation factors (VIFs) for all models were low (≈ 1.0–1.2), indicating negligible multicollinearity.

To examine associations among academic performance, working memory, and daytime sleepiness, we fit a priori moderation models including both WM indicators (CTB Total, CTB Average RT), PDSS, and their cross-product terms with PDSS (CTB Total × PDSS; CTB RT × PDSS). Sex and school level were entered as covariates in all models. Each regression tested two interaction terms between working memory and daytime sleepiness. The general model specification was

(1) $$Grade=\beta 0+\beta 1\left(CTBTotal\right)+\beta 2\left(CTBRT\right)+\beta 3\left(PDSS\right)+\beta 4\left(CTBTotal\times PDSS\right)+\beta 5(CTBRT\times PDSS)+\beta 6(Sex)+\beta 7(SchoolLevel)+\varepsilon$$

Because continuous predictors were z-standardized prior to modelling, the reported unstandardized coefficients (B) for CTB Total, CTB Average RT, and PDSS can be interpreted as the expected change in grade units associated with a 1 SD increase in the predictor. We specified a single, theory-driven model to preserve comparability across outcomes and avoid data-driven specification search; sensitivity models entering CTB Total and CTB Average RT separately produced the same qualitative pattern of *B* and *CI*s. An exploratory model that additionally included the CTB Total × CTB Average RT term (WM × WM interaction) showed a near-zero coefficient (|*B*| < 0.03, 95% bootstrap *CI*s spanning zero) and negligible change in adjusted *R*^2^ (< 0.002); given the weak empirical association between the WM indicators and no a priori rationale, this term was not retained in final tables.

Equivalent models were estimated for each academic outcome (Mathematics, Russian Language, Literature, and the composite Average Grade). Statistical significance was assessed using nonparametric bootstrapping with 5000 replications to obtain robust estimates of regression coefficients. Because the interaction term was not significant in any models, no mediation analyses were conducted.

To explore potential differences by the severity of daytime sleepiness, additional regression models were estimated separately for subgroups with normal (PDSS < 16) and high (PDSS ≥ 16) levels of sleepiness. The significance threshold was set at *p* < 0.05. Independent *t*-tests and χ^2^ tests were used to examine baseline differences between these subgroups.

Post hoc power analysis indicated that the available sample size provided > 0.80 power to detect moderate interaction effects (*f*^2^ ≈ 0.06) but might have been underpowered for smaller effects.

## 5. Conclusions

Although the hypothesized statistical moderation was not confirmed, subgroup differences indicate that daytime sleepiness can attenuate the benefits of strong working memory, particularly in cognitively demanding subjects such as mathematics. These findings support the importance of assessing both cognitive and physiological factors when evaluating students’ academic risk. Sleepiness should be treated not merely as a background variable but as a potential barrier to learning—especially during adolescence, when cognitive demands rise and sleep quality tends to decline. Targeted interventions aimed at reducing sleep debt and improving alertness could enhance the educational outcomes of students who might otherwise underperform despite adequate cognitive skills.

## Figures and Tables

**Table 1:** Distribution of participants by age and gender.

School Level	Male (*N*)	Female (*N*)	Total (*N*)
Middle school (11–15 y)	193	170	363
High school (16–17 y)	98	140	238
Total	291	310	601

**Table 2:** Descriptive statistics for working memory and academic variables (*N* = 601).

Variable	Mean	SD	Median	SE
Average Grade	4.05	0.58	4	0.02
Language Grade	3.93	0.64	4	0.03
Mathematics Grade	3.94	0.75	4	0.03
Literature Grade	4.29	0.64	4	0.03
CTB Total Score	4.72	1.95	5	0.08
CTB Average RT	4654.30	1513.19	4519	61.75
PDSS	14.07	6.45	14	0.26

**Table 3:** Correlations between academic performance, cognitive indicators, and daytime sleepiness (*N* = 601).

Variable	CTB Total Score	CTB Average RT	PDSS
Average Grade	0.12 **	−0.06	−0.11 **
Language Grade	0.11 **	−0.06	−0.08
Mathematics Grade	0.14 ***	−0.10 *	−0.11 **
Literature Grade	0.05	0.01	−0.11 **

Note. * *p* < 0.05, ** *p* < 0.01, *** *p* < 0.001; PDSS = Pediatric Daytime Sleepiness Scale; CTB = Corsi Block-Tapping Test; RT = reaction time.

**Table 4:** Regression Coefficients for the Moderation Model Predicting Average Grade.

Predictor	*B*	*SE*	*t*	*p*
Intercept	3.85	0.04	105.26	<0.001
CTB Total Score	0.06	0.02	2.58	0.010
PDSS	−0.10	0.02	−4.34	<0.001
CTB Average RT	−0.06	0.02	−2.68	0.008
Sex (Female)	0.33	0.05	7.05	<0.001
School Level (High)	0.09	0.05	1.94	0.052
CTB Total × PDSS	0.01	0.02	0.29	0.772
PDSS × CTB Average RT	0.03	0.02	1.24	0.215

Note. All continuous predictors were standardized. *B* = unstandardized regression coefficient; *SE* = standard error; RT = reaction time; PDSS = Pediatric Daytime Sleepiness Scale; CTB = Corsi Block-Tapping Test.

**Table 5:** Regression Coefficients for the Moderation Model Predicting Mathematics Grade.

Predictor	*B*	*SE*	*t*	*p*
Intercept	3.66	0.05	78.06	<0.001
CTB Total Score	0.07	0.03	2.48	0.013
PDSS	−0.12	0.03	−4.18	<0.001
CTB Average RT	−0.10	0.03	−3.30	0.001
Sex (Female)	0.32	0.06	5.33	<0.001
School Level (High)	0.29	0.06	4.66	<0.001
CTB Total × PDSS	−0.01	0.03	−0.39	0.701
PDSS × CTB Average RT	0.01	0.03	0.21	0.835

Note. All continuous predictors were standardized. *B* = unstandardized regression coefficient; *SE* = standard error; RT = reaction time; PDSS = Pediatric Daytime Sleepiness Scale; CTB = Corsi Block-Tapping Test.

**Table 6:** Regression Coefficients for the Moderation Model Predicting Language Grade.

Predictor	*B*	*SE*	*t*	*p*
Intercept	3.72	0.04	91.11	<0.001
CTB Total Score	0.07	0.03	2.68	0.008
PDSS	−0.09	0.03	−3.37	<0.001
CTB Average RT	−0.07	0.03	−2.68	0.008
Sex (Female)	0.38	0.05	7.38	<0.001
School Level (High)	0.03	0.05	0.59	0.554
CTB Total × PDSS	0.00	0.03	0.01	0.992
PDSS × CTB Average RT	0.04	0.03	1.58	0.115

Note. All continuous predictors were standardized. *B* = unstandardized regression coefficient; *SE* = standard error; RT = reaction time; PDSS = Pediatric Daytime Sleepiness Scale; CTB = Corsi Block-Tapping Test.

**Table 7:** Regression Coefficients for the Moderation Model Predicting Literature Grade.

Predictor	*B*	*SE*	*t*	*p*
Intercept	4.17	0.04	99.97	<0.001
CTB Total Score	0.04	0.03	1.37	0.170
PDSS	−0.09	0.03	−3.41	<0.001
CTB Average RT	−0.02	0.03	−0.71	0.478
Sex (Female)	0.28	0.05	5.34	<0.001
School Level (High)	−0.04	0.05	−0.71	0.478
CTB Total × PDSS	0.03	0.03	1.21	0.228
PDSS × CTB Average RT	0.04	0.03	1.49	0.138

Note. All continuous predictors were standardized. *B* = unstandardized regression coefficient; *SE* = standard error; RT = reaction time; PDSS = Pediatric Daytime Sleepiness Scale; CTB = Corsi Block-Tapping Test.

**Table 8:** Baseline Characteristics of Students with Normal (PDSS < 16) vs. High (PDSS ≥ 16) Daytime Sleepiness.

Variable	Normal Sleepiness	High Sleepiness	Test Statistic	*p*-Value	Effect Size
Age, *M* (*SD*)	13.99 (2.00)	14.45 (1.96)	*t*(517.0) = −2.76	0.006	*d* = −0.23
CTB Total Score, *M* (*SD*)	4.77 (1.95)	4.63 (1.95)	*t*(509.7) = 0.86	0.393	*d* = 0.07
CTB Average RT, *M* (*SD*)	4763.69 (1526.33)	4488.61 (1482.72)	*t*(519.8) = 2.20	0.028	*d* = 0.14
Female, *N* (%)	162 (44.8%)	148 (61.9%)	χ^2^(1) = 16.32	<0.001	*V* = 0.17
High school, *N* (%)	127 (35.1%)	111 (46.4%)	χ^2^(1) = 7.30	0.007	*V* = 0.11

**Table 9:** Regression Coefficients for the Moderation Model Predicting Average Grade in Normal and High Daytime Sleepiness.

Daytime Sleepiness	Normal (PDSS < 16, *N* = 362)				High (PDSS ≥ 16, *N* = 239)			
Predictor	*B*	*SE*	*t*	*p*	*B*	*SE*	*t*	*p*
Intercept	3.91	0.06	64.11	<0.001	3.85	0.09	43.01	<0.001
CTB Total Score	0.10	0.05	2.22	0.027	0.12	0.08	1.57	0.117
PDSS	−0.06	0.05	−1.04	0.300	−0.15	0.06	−2.43	0.016
CTB Average RT	−0.07	0.04	−1.66	0.098	−0.09	0.08	−1.10	0.271
Sex (Female)	0.27	0.06	4.50	<0.001	0.42	0.07	5.74	<0.001
School Level (High)	0.10	0.06	1.57	0.117	0.05	0.08	0.62	0.537
CTB Total × PDSS	0.06	0.05	1.18	0.237	−0.06	0.07	−0.88	0.378
PDSS × CTB Average RT	0.00	0.05	0.08	0.941	0.05	0.07	0.70	0.485

Note. All continuous predictors were standardized. *B* = unstandardized regression coefficient; *SE* = standard error; RT = reaction time; PDSS = Pediatric Daytime Sleepiness Scale; CTB = Corsi Block-Tapping Test.

**Table 10:** Regression Coefficients for the Moderation Model Predicting Mathematics Grade in Normal and High Daytime Sleepiness.

Daytime Sleepiness	Normal (PDSS < 16, *N* = 362)				High (PDSS ≥ 16, *N* = 239)			
Predictor	*B*	*SE*	*t*	*p*	*B*	*SE*	*t*	*p*
Intercept	3.73	0.08	48.15	<0.001	3.67	0.12	31.27	<0.001
CTB Total Score	0.12	0.06	2.01	0.045	0.07	0.10	0.66	0.511
PDSS	−0.05	0.07	−0.80	0.426	−0.15	0.08	−1.88	0.061
CTB Average RT	−0.11	0.06	−2.00	0.046	−0.05	0.10	−0.45	0.656
Sex (Female)	0.29	0.08	3.78	<0.001	0.36	0.10	3.73	<0.001
School Level (High)	0.29	0.08	3.63	<0.001	0.25	0.10	2.48	0.014
CTB Total Score × PDSS	0.03	0.07	0.51	0.608	−0.02	0.09	−0.18	0.855
PDSS × CTB Average RT	−0.01	0.07	−0.16	0.875	−0.04	0.09	−0.46	0.644

Note. All continuous predictors were standardized. *B* = unstandardized regression coefficient; *SE* = standard error; RT = reaction time; PDSS = Pediatric Daytime Sleepiness Scale; CTB = Corsi Block-Tapping Test.

**Table 11:** Regression Coefficients for the Moderation Model Predicting Language Grade in Normal and High Daytime Sleepiness.

Daytime Sleepiness	Normal (PDSS < 16, *N* = 362)				High (PDSS ≥ 16, *N* = 239)			
Predictor	*B*	*SE*	*t*	*p*	*B*	*SE*	*t*	*p*
Intercept	3.73	0.07	54.06	< 0.001	3.79	0.10	39.04	< 0.001
CTB Total Score	0.12	0.05	2.32	0.021	0.16	0.08	1.97	0.051
PDSS	−0.08	0.06	−1.26	0.208	−0.17	0.07	−2.47	0.014
CTB Average RT	−0.08	0.05	−1.58	0.115	−0.13	0.08	−1.51	0.131
Sex (Female)	0.33	0.07	4.88	< 0.001	0.48	0.08	6.04	< 0.001
School Level (High)	0.09	0.07	1.26	0.211	−0.09	0.08	−1.14	0.256
CTB Total × PDSS	0.07	0.06	1.27	0.206	−0.09	0.07	−1.28	0.204
PDSS × CTB Average RT	0.01	0.06	0.22	0.830	0.09	0.08	1.14	0.257

Note. All continuous predictors were standardized. *B* = unstandardized regression coefficient; *SE* = standard error; RT = reaction time; PDSS = Pediatric Daytime Sleepiness Scale; CTB = Corsi Block-Tapping Test.

**Table 12:** Regression Coefficients for the Moderation Model Predicting Literature Grade in Normal and High Daytime Sleepiness.

Daytime Sleepiness	Normal (PDSS < 16, *N* = 362)				High (PDSS ≥ 16, *N* = 239)			
Predictor	*B*	*SE*	*t*	*p*	*B*	*SE*	*t*	*p*
Intercept	4.26	0.07	62.79	<0.001	4.09	0.11	38.96	<0.001
CTB Total Score	0.07	0.05	1.33	0.184	0.13	0.09	1.47	0.143
PDSS	−0.04	0.06	−0.60	0.547	−0.13	0.07	−1.84	0.068
CTB Average RT	−0.03	0.05	−0.59	0.559	−0.08	0.09	−0.92	0.357
Sex (Female)	0.19	0.07	2.86	0.005	0.43	0.09	4.94	<0.001
School Level (High)	−0.08	0.07	−1.19	0.235	−0.01	0.09	−0.13	0.896
CTB Total × PDSS	0.08	0.06	1.32	0.189	−0.07	0.08	−0.88	0.382
PDSS × CTB Average RT	0.01	0.06	0.16	0.871	0.11	0.08	1.26	0.210

Note. All continuous predictors were standardized. *B* = unstandardized regression coefficient; *SE* = standard error; RT = reaction time; PDSS = Pediatric Daytime Sleepiness Scale; CTB = Corsi Block-Tapping Test.

## Data Availability

Data are available from the authors upon reasonable request.

## Supplementary Materials

The following supporting information can be downloaded at https://www.mdpi.com/article/10.3390/clockssleep7040057/s1, Table S1: Distribution of available quarterly grades by school level; Table S2: Descriptive statistics of grades and task performance by school level.

## Abbreviations

The following abbreviations are used in this manuscript:

PDSS	Pediatric Daytime Sleepiness Scale
CTB	Corsi Block-Tapping Test
RT	Reaction time
IQR	Interquartile Range
