# Supplementary material for: Does Daytime Sleepiness Moderate the Relationship Between Working Memory and Academic Performance in Schoolchildren? A Pilot Study
Source: Clocks Sleep. 2025 Oct 8;7(4):57. doi: 10.3390/clockssleep7040057 (PMC12550925; doi:10.3390/clockssleep7040057)
Supplement: Supplementary file 1 [file clockssleep-07-00057-s001.zip › clockssleep-3713807-supplementary.pdf]

Supplementary Table S1. Distribution of available quarterly grades by school level

School level	1 quarterly grade	2 quarterly grades	Total
Middle school	n = 325	n = 38	363
High school	n = 217	n = 21	238
Total	n = 542	n = 59	601

Supplementary Table S2. Descriptive statistics of grades and task performance by school level

Variable	Middle school (*N* = 363)	High school (*N* = 238)
PDSS total score	13.51 (6.83)	14.92 (5.73)
CTB TotalScore	4.39 (1.89)	5.21 (1.94)
Mathematics grade	3.81 (0.69)	4.14 (0.78)
Russian Language grade	3.89 (0.69)	3.98 (0.57)
Literature grade	4.29 (0.70)	4.29 (0.55)
